# Double lumen concentric frontal sinus irrigation device

**DOI:** 10.1308/rcsann.2024.0083

**Published:** 2025-10-21

**Authors:** J Kirk, S Arman, R Gohil

**Affiliations:** NHS Lothian, UK

## Background

Trephination is a surgical technique used to address acute frontal sinusitis, either in place of or as an adjunct to endoscopic approaches to the frontal sinus.^1,2^ This technique is a surgical option when addressing complications of acute sinusitis, e.g. intracranial collection. Here, we describe a simple technique to create a device with the purpose of irrigating the frontal sinus following trephination.

## Technique

A size 4 paediatric endotracheal tube (ET) (Portex, Smiths Medical, USA; 4.0mm internal diameter and 5.5mm external diameter) is used and the anaesthetic connector is removed.

**Figure 1:**
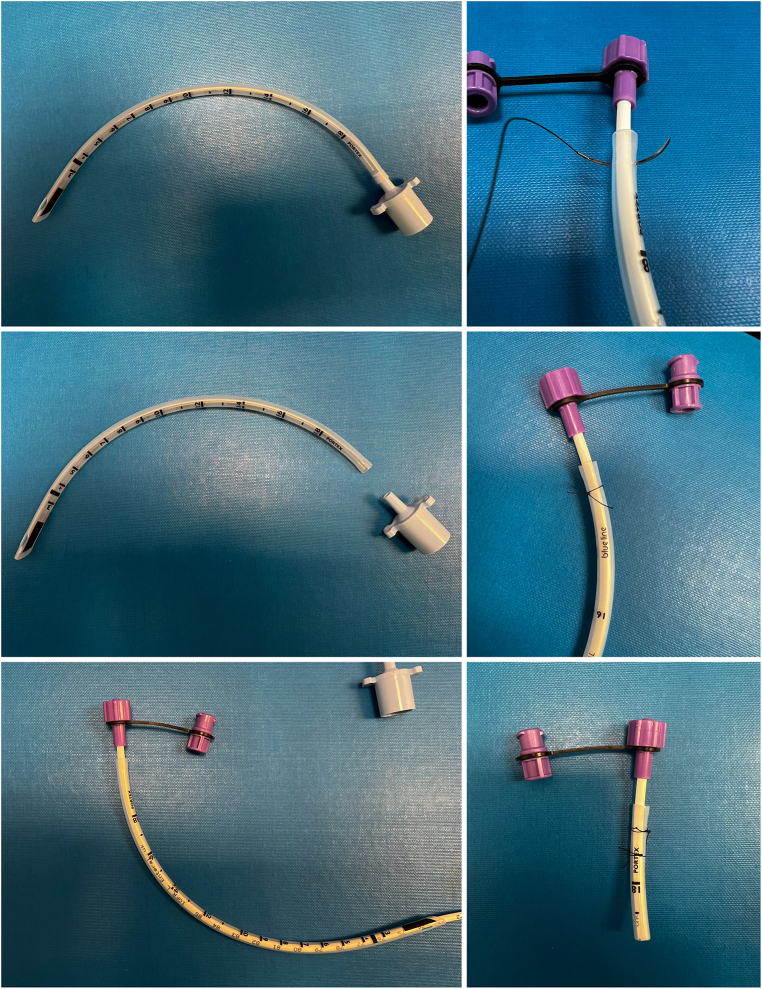
Six steps showing preparation of the device as described. This example uses an endotracheal tube with a 4mm internal diameter (5.5mm external diameter) and a nasogastric tube with a 2.1mm internal diameter (3.33mm external diameter).

A complementary 10Fr nasogastric (NG) feeding tube (Nutricare, GBUK Group Ltd, UK; 10Fr × 92cm) is passed through the ET tube and both cut to the desired length (∼4–5cm).

The tubes are held in position relative to each other with the aid of one or two silk sutures (4-0 or equivalent) (Figure 1). The drain is passed percutaneously into the trephination site and further secured with a suture.

The device is used to deliver saline through the NG tube using a compatible syringe.

**Figure 2:**
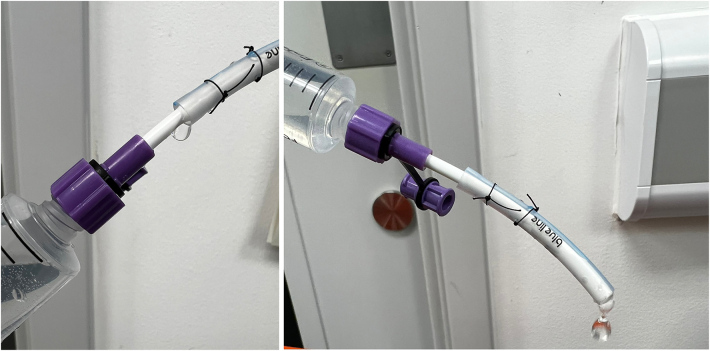
In the first image, the exit is occluded, causing saline to flow back via the larger endotracheal lumen and exit at the proximal end. In the second example, saline can flow freely from the nasogastric lumen.

If the frontal recess drainage pathway is still occluded, the limited volume in the sinus will cause backflow of saline through the ET lumen, and the user will see saline exit the tube at the proximal end of the drain (Figure 2). When the occlusion is resolved, saline will pass freely through the NG lumen into the sinus, descend the frontal recess and enter the pharynx. At this point, the patient can taste the saline and provide feedback.

## Discussion

This technique enables safe and effective irrigation of the frontal sinus with commonly available tools, simply modified. It provides immediate feedback to the user, reducing the length of time the drain or irrigation device remains in situ. Care must be taken to avoid the use of this device in instances where the posterior table of frontal sinus is dehiscent.

## Conflict of interest

The authors have no conflicts of interest to declare.
